# Exploring the therapeutic potency of cryptotanshinone in cervical cancer: a multi-omics and network pharmacology approach

**DOI:** 10.3389/fgene.2024.1435132

**Published:** 2024-11-27

**Authors:** Zenghong Lu, Gangfeng Zhu, Xiaofei Feng, Yi Xiang, Cixiang Chen, Huiting Yuan, Zhixing Chen

**Affiliations:** ^1^ Department of Oncology, The First Affiliated Hospital, Gannan Medical University, Ganzhou, China; ^2^ First Clinical Medical College, Gannan Medical University, Ganzhou, China; ^3^ Department of Gastroenterology, The First Affiliated Hospital, Gannan Medical University, Ganzhou, China

**Keywords:** cryptotanshinone, cervical cancer, multi-omics analysis, network pharmacology, bioinformatics, prognostic biomarkers

## Abstract

**Introduction:**

Cervical cancer remains a significant challenge in oncology with an escalating demand for novel therapeutic strategies that can navigate the complexities of its pathophysiology. This study elucidated the antineoplastic effects of cryptotanshinone, a derivative of danshen (*Salvia miltiorrhiza*), a herb widely utilized in traditional Chinese medicine practices.

**Methods:**

Employing a comprehensive multi-omics approach, including transcriptomic, proteomic, and bioinformatics analyses, we investigated the potential effects of cryptotanshinone on cervical cancer through data mining and computational analysis.

**Results and Discussion:**

Our results demonstrated that the potential of cryptotanshinone to disrupted cancer cell proliferation and induced apoptosis may be ascribed to its modulation of gene expression and interaction with specific protein networks. Furthermore, network pharmacology and pathway enrichment analyses identified critical hubs and signaling pathways, suggesting a multi-targeted mechanism of action. Furthermore, the establishment of a prognostic model, which is founded upon differentially expressed genes linked to cryptotanshinone treatment, underscores its promising role as both a prognostic biomarker and a therapeutic agent. These insights pave the way for the integration of cryptotanshinone into therapeutic regimens, offering a promising avenue for enhancing the efficacy of cervical cancer treatment and patient outcomes.

## 1 Introduction

Cervical cancer remains a critical public health issue and is characterized by significant global prevalence and mortality rates. This malignancy, primarily manifesting as squamous cell carcinoma and adenocarcinoma, originates in the cervix and lower part of the uterus. Human Papillomavirus (HPV) infection has been definitively recognized as the primary contributory factor leading to the development of cervical cancer, underscoring the importance of preventive measures such as regular screening and HPV vaccination (Lei et al., 2020). The severity of the disease is particularly pronounced in low- and middle-income economies, where limited availability of preventive healthcare and treatment options often exacerbates the situation. It is crucial to decrease the impact of cervical cancer by making progress in comprehending the biological processes involved and creating successful treatment choices. The advancement of innovative diagnostic and therapeutic methodologies is crucial for enhancing patient outcomes as early detection significantly improves prognosis (Sundstrom and Elfstrom, 2020).

Danshen (*Salvia miltiorrhiza*), a widely used herb in traditional Chinese medicine, and its bioactive compound, cryptotanshinone, have garnered attention for their therapeutic potential in oncology, including cervical cancer treatment (Jin et al., 2021). Historically utilized for the therapy of cardiovascular and cerebrovascular diseases, Danshen exhibits anti-inflammatory, antioxidant, and antitumor properties. Cryptotanshinone has been the subject of research due to its potential in inhibiting the proliferation of cancer cells and triggering apoptosis. (Zhang et al., 2020). The pharmacological effects of danshen and cryptotanshinone highlight their relevance in cancer research and offer a promising avenue for novel treatment strategies that target the complex pathophysiology of cervical cancer (Liu et al., 2020). Their incorporation into cancer treatment regimens underscores the integration of traditional medicine into modern oncological practices, potentially enhancing therapeutic outcomes and patient quality of life (Li et al., 2021).

In our preliminary investigations using the HERB and SymMap databases, we identified Danshen and Lei Gong Teng as potential botanical agents implicated in the etiology and progression of cervical cancer. These findings highlight the intricate interplay between specific herbal compounds and the pathophysiological mechanisms of cervical cancer and suggest a promising direction for future research. This exploration of traditional medicinal herbs using contemporary bioinformatic approaches provides novel insights into their potential therapeutic roles, paving the way for innovative treatment strategies against cervical cancer.

The burgeoning resistance to conventional chemotherapeutic agents and adverse side effects associated with current cervical cancer treatments underscore the urgent need for innovative therapeutic strategies. Natural compounds, such as danshen and cryptotanshinone have emerged as promising candidates owing to their multi-targeted therapeutic potential and low toxicity profiles. Their capacity to regulate critical signaling pathways implicated in cancer cell proliferation, apoptosis, and metastasis positions them as viable complements or alternatives to traditional treatments (Wang et al., 2024; Song et al., 2023). This paradigm shift toward incorporating phytochemicals into cancer management could significantly improve treatment outcomes and patient quality of life, requiring further exploration of their clinical applicability.

Network drug analysis represents a cutting-edge approach in cancer research that leverages the power of systems biology to unravel the complex interactions within the cellular networks exploited by cancer cells (Theodoris et al., 2023). This approach aids in the discovery of new drug targets and understanding of drug mechanisms by examining the interconnected pathways and genetic networks that are modified in cancer.Moreover, it offers the potential to repurpose existing drugs for cervical cancer treatment by revealing previously unrecognized anticancer activities within their pharmacological profiles. This innovative strategy holds promise for accelerating the development of more effective and targeted therapies for cervical cancer, thereby enhancing the precision of treatment interventions.

This research aimed to investigate the therapeutic efficacy and underlying mechanisms of danshen and cryptotanshinone in treating cervical cancer. By focusing on these natural compounds, we aimed to elucidate their impact on cancer cell biology, their potential to inhibit tumor growth, and their ability to improve patient outcomes.

## 2 Materials and methods

### 2.1 Data download

Utilizing the TCGAbiolinks package in R (Colaprico et al., 2016) we retrieved datasets specific to cervical endocervical adenocarcinoma and squamous cell carcinoma (CESC) from The Cancer Genome Atlas (TCGA-CESC). This data was used as the primary test set. Following the exclusion of samples that lacked comprehensive clinical details, the final count included 306 CESC samples and three normal controls, all sequenced in count format. These data have been standardized by FPKM per thousand bases, and the relevant clinical information was obtained from the UCSC Xena database (Goldman et al., 2020) (https://xena.ucsc.edu/). For additional details, please see Table 1.

**TABLE 1:** Overall baseline data sheet.

Characteristics	Overall
Pathologic T stage, n (%)	
T1	142 (53.8%)
T2	74 (28.0%)
T3	21 (8.0%)
T4	10 (3.8%)
TX	17 (6.4%)
Pathologic N stage, n (%)	
N0	136 (51.5%)
N1	62 (23.5%)
NX	66 (25.0%)
Pathologic M stage, n (%)	
M0	116 (44.8%)
M1	11 (4.2%)
MX	132 (51.0%)
Age, n (%)	
≤ 50	189 (61.2%)
>50	120 (38.8%)

For validation purposes, we employed the GEOquery package (Davis and Meltzer, 2007; Barrett et al., 2013) to access cervical cancer datasets GSE7803 (Zhai et al., 2007) and GSE9750 (Scotto et al., 2008) from the GEO database (https://www.ncbi.nlm.nih.gov/geo/). These datasets consisted of cervical tissue samples analyzed on GPL96 microarrays. The GSE7803 dataset included 21 CESC and 10 normal samples, whereas GSE9750 comprised 33 CESC and 24 normal samples. Comprehensive sample details are available in Supplementary Table S1.

To address batch effects during the integration of TCGA-CESC and GEO datasets, we utilized the sva package (Leek et al., 2012) specifically utilizing the ComBat method within an empirical Bayes framework. We initially used Principal Component Analysis (PCA) to detect potential discrepancies in the datasets, enabling us to visually assess batch effects before integration. After correction with ComBat, PCA was reapplied, confirming effective batch effect removal through clearer clustering of samples. This resulted in a harmonized dataset comprising 54 CESC and 34 normal samples. Subsequently, the limma package (Ritchie et al., 2015) was used to standardize this combined dataset, including probe annotations and processing steps. The standardized dataset was then visualized in two- or three-dimensional PCA plots, providing a reduced-dimensionality perspective that demonstrated successful integration.

### 2.2 Cryptotanshinone target prediction

Initially, an exploration of Cryptotanshinone’s potential target genes was conducted by accessing the PubChem database (Kim et al., 2021) (https://pubchem.ncbi.nlm.nih.gov), a repository rich in chemical data pertinent to drug discovery. The search term “Cryptotanshinone” yielded 46 cryptotanshinone-related genes (CTSRGs). Further predictive analysis was performed using the SwissTargetPrediction tool (Daina et al., 2019) (http://swisstargetprediction.ch/), which suggested an additional 100 CTSRGs. Complementing these methods, the DGIdb database (Freshour et al., 2021) (https://dgidb.org/), which catalogs potential drug-gene interactions, was queried using “Cryptotanshinone” as the keyword, identifying 14 unique CTSRGs. A comprehensive set of 133 CTSRGs was assembled from these sources, and their interactions were visualized using a network map created using Cytoscape software (Shannon et al., 2003).

### 2.3 Cryptotanshinone-related differentially expressed genes associated with cervical cancer

Analysis of the TCGA-CESC dataset, which segregates samples into CESC and normal controls, was conducted using the DESeq2 package (Love et al., 2014) Differentially expressed genes (DEGs) were identified using strict criteria, with |logFC| > 3.0 and adjusted *p*-value <0.05. Upregulated genes exhibited logFC >3.0 and adj. *p* < 0.05, while downregulated genes presented with |logFC| < −3.0 and adj. *p* < 0.05. The Benjamini–Hochberg method was applied for *p*-value correction. These differential expression results were graphically depicted using the ggplot2 package in R.

To discern Cryptotanshinone-related differentially expressed genes (CTSRDEGs) pertinent to cervical cancer, DEGs from TCGA-CESC dataset that met the criteria of |logFC| > 3.0 and *P*-adj <0.05 were intersected with the CTSRGs, and the intersections were visualized using a Venn diagram. Using the pheatmap package in R to create a heatmap of the CTSRDEGs further elucidates the gene expression changes associated with Cryptotanshinone expression in cervical cancer.

### 2.4 Protein–protein interaction (PPI) network and hub gene screening

The PPI network encompasses a complex web of interacting proteins that play critical roles in various biological functions including signal transduction, gene regulation, and essential life processes like metabolic pathways and cell cycle control. Analyzing these interactions provides profound insights into protein functions, biological signaling mechanisms, and metabolic processes under specific physiological conditions, including disease states. For constructing the PPI network related to CTSRDEGs, we utilized the STRING database (Szklarczyk et al., 2019) (https://string-db.org/) to map out both known and predicted protein interactions, setting a minimum confidence score threshold of 0.40. Regions within the PPI network demonstrating high connectivity often indicate the presence of protein complexes linked to specific biological functions. We employed several algorithms via the CytoHubba (Chin et al., 2014) plugin in Cytoscape to determine the centrality of nodes within the network: Maximal Clique Centrality (MCC), Degree, Maximum Neighborhood Component (MNC), Edge-Percolated Component (EPC), and Closeness (Yang et al., 2019). The top 10 CTSRDEGs were identified based on their network scores, and the overlap of results from these algorithms highlighted key hub genes associated with cryptotanshinone.

### 2.5 Enrichment analysis of gene ontology (GO) and kyoto encyclopedia of genes and genomes (KEGG) pathway

Functional enrichment analysis is pivotal for understanding the roles of genes within biological contexts. GO analysis (Mi et al., 2019) and the Kyoto Encyclopedia of Genes and Genomes (KEGG) (Kanehisa and Goto, 2000) are instrumental in elucidating the biological processes, cellular components, molecular functions, and pathway interactions of genes. We used the clusterProfiler software package (Engebretsen and Bohlin, 2019) to conduct a comprehensive enrichment analysis of the hub genes associated with cryptotanshinone. Criteria for significant enrichment included a *p*-value of less than 0.05 and an FDR (*q* value) of less than 0.25. Results were visualized using Cytoscape, creating a network map that integrates cryptotanshinone, its related hub genes, significant GO terms, and enriched KEGG pathways. Furthermore, pathway illustrations based on KEGG analysis were generated using the Pathview package (Luo and Brouwer, 2013), providing a visual representation of the pathways involved.

### 2.6 Differential expression verification and receiver operating characteristic (ROC) curve analysis of cryptotanshinone-related hub genes

We investigated the expression differences of genes responsive to cryptotanshinone between cervical cancer samples (CESC) and normal controls across both the TCGA-CESC and Combined GEO datasets. Use the Mann-Whitney U test to analyze expression discrepancies, resulting in a visual comparison of gene expression levels between groups. Subsequent analysis of the diagnostic capabilities of these genes was performed using the pROC package in R to plot ROC curves and determine the Area Under Curve (AUC) values for each gene. The diagnostic performance was interpreted based on AUC values, where an AUC close to 1 indicates excellent diagnostic accuracy. Specifically, AUC values ranging from 0.5 to 0.7 indicates a low diagnostic accuracy, while those ranging from 0.7 to 0.9 suggest a moderate level of accuracy, and values exceeding 0.9 reflect high levels of accuracy.

### 2.7 Construction of prognostic risk model and prognostic analysis of cervical cancer

In TCGA-CESC dataset, a prognostic risk model was developed using the survival package in R (Therneau, 2023). Initial univariate Cox regression analyses identified cryptotanshinone-sensitive DEGs (CTSRDEGs) with a significant impact on prognosis (*p* < 0.10). These genes underwent additional Least Absolute Shrinkage and Selection Operator (LASSO) regression, utilizing the glmnet package in R with a Cox family parameter, to refine the model and enhance its predictive robustness (Engebretsen and Bohlin, 2019). The LASSO regression, which penalizes the regression model by incorporating a lambda parameter to reduce overfitting, generated a RiskScore computed as the summation of gene coefficients multiplied by their corresponding mRNA expressions. The LASSO RiskScore was calculated as follows:

$$\text{risk}Score=\sum_{i}Coefficient\;\left({gene}_{i}\right)*mRNA\;Expression\;\left({gene}_{i}\right)$$

The prognostic model underwent further validation through multivariate Cox regression analysis, incorporating the identified CTSRDEGs from the LASSO model. The impact of these genes on survival was graphically represented in a Forest Plot. Utilizing the median LASSO RiskScore, cervical cancer samples were categorized into high- and low-risk groups.

The comparison of survival between these groups was conducted using the Kaplan-Meier curve analysis with the survival package (Rich et al., 2010), and a time-dependent ROC curve was generated with the Surviroc package to assess the precision of the prognostic model in forecasting 1-, 3-, and 5-year survival rates (Park et al., 2004). The AUC values derived from the ROC analysis provided a quantitative measure of the model’s predictive accuracy, where higher values indicate superior prognostic performance.

### 2.8 Validation of cervical cancer prognostic risk models

The relationship between the LASSO RiskScore expression and clinical outcomes was investigated through univariate Cox regression analysis, incorporating the LASSO RiskScore alongside age and three clinical staging parameters (T stage, N stage, and M stage). The outcomes of both univariate and multivariate Cox regression analyses were graphically depicted using forest plots to elucidate the impact of LASSO RiskScore and other clinical factors. A nomogram (Wu et al., 2020), constructed with the rms package, represented the multifactorial relationships and predicted the 1-, 3-, and 5-year survival probabilities based on variables included in the multivariate Cox regression model.

The model’s predictive performance was evaluated using a calibration curve, which compares the actual outcomes with those predicted by the model across various scenarios. This curve was crucial for assessing the precision and reliability of the prognostic model over 1-, 3-, and 5-year periods, as indicated by the nomogram. Additionally, the decision curve analysis (DCA), implemented with the ggDCA package, was utilized to assess the clinical utility of the nomogram predictions for these time frames.

### 2.9 Analysis of differential expression and correlation in risk groups

The TCGA-CESC dataset was divided into high-risk and low-risk groups based on the median LASSO RiskScore. The risk stratification was similarly utilized for the combined GEO dataset using the LASSO RiskScore calculated from the risk coefficients. Further investigation was performed to examine the variation in gene expression related to the prognostic risk model within both high and low-risk groups of TCGA-CESC and combined GEO datasets. Expression comparison graphs were created to visualize these differences. Correlations among the genes related to the prognostic risk model in the TCGA-CESC and combined GEO datasets were analyzed using the Spearman correlation method. The correlation chord graphs produced by the igraph and ggraph packages were used to depict the associations among gene expression levels. The strength of the correlation was classified based on the correlation coefficient (r value): values below 0.3 indicated negligible to weak correlation, between 0.3 and 0.5 indicated weak correlation, between 0.5 and 0.8 suggested moderate correlation, and above 0.8 indicated strong correlation.

### 2.10 Immuno-infiltration analysis of cervical cancer

Utilizing the CIBERSORT algorithm (Newman et al., 2015), which is grounded in linear support vector regression, we decomposed the transcriptomic data to assess immune cell compositions and abundances within mixed cellular contexts. Applying the LM22 gene signature, we processed the data to retain only samples with nonzero immune cell fractions, leading to the derivation of the immune cell infiltration matrix for the TCGA-CESC dataset. The ggplot2 package in R was utilized to visualize variations in immune cell profiles between CESC and normal samples, highlighting significant variances in LM22-defined immune cell types. Spearman’s rank correlation was employed to both assess inter-immune cell correlations and link these cells with model genes, identifying statistically significant relationships (*p* < 0.05). Visualization of these correlations was achieved through correlation heatmaps and bubble plots, generated with the pheatmap and ggplot2 packages, respectively.

### 2.11 Analysis of immune profiles in high vs. low-risk cervical cancer groups

Through Single-Sample Gene-Set Enrichment Analysis (ssGSEA) (Xiao et al., 2020), we quantified the degree of immune cell infiltration, encompassing activated CD8 T cells, dendritic cells, and other immune subsets, within the CESC samples of the TCGA-CESC dataset. These measurements provided a relative abundance score for each immune type, forming the basis for constructing an immune cell invasion matrix. Comparative analyses of immune cell abundance between high and low-risk groups were illustrated utilizing ggplot2, with significant immune cell variances noted for further analysis. Spearman’s correlation was again utilized to explore both intra-immune cell relationships and their associations with model genes. The findings were presented through heatmaps and bubble maps, crafted using pheatmap and ggplot2.

### 2.12 Statistical analysis

All analytical procedures were executed in R software (Version 4.2.2). Statistical significance for data with a normal distribution was assessed by employing the Student’s t-test to compare two continuous variables. For data not following normal distribution, the Mann-Whitney U test, or the Wilcoxon Rank Sum Test, was applied. Multiple group comparisons were conducted using the Kruskal–Wallis test. Spearman’s correlation coefficient was computed to determine the relationships among diverse biomolecules. Statistical significance was set at a bilateral *p*-value of less than 0.05, unless specified otherwise.

## 3 Results

### 3.1 Technology roadmap and target prediction of cryptotanshinone

**FIGURE 1:**
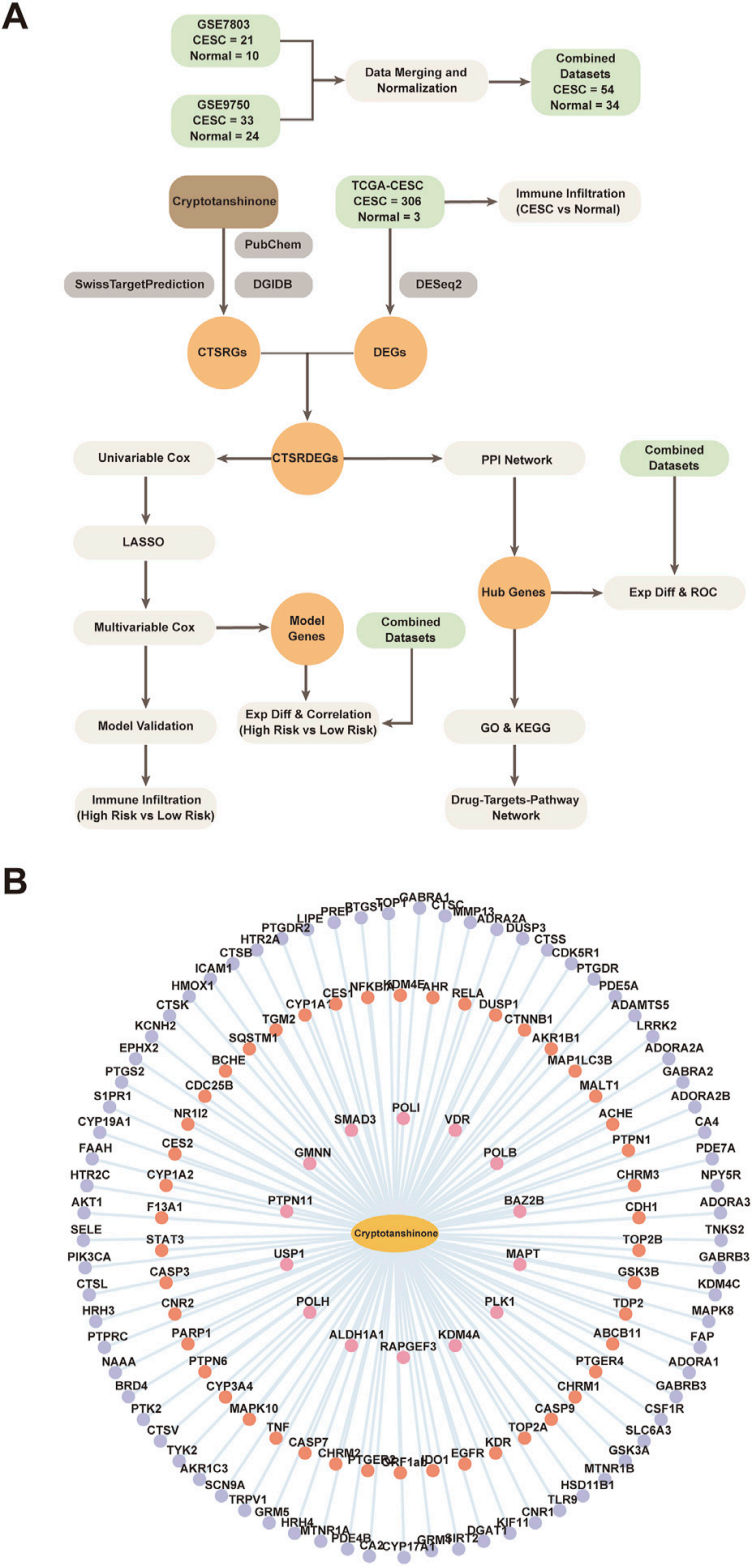
Comprehensive Analysis and Interaction Network of Cryptotanshinone-Related Differentially Expressed Genes (CTSRDEGs). **(A)** Flow Chart for analysis of CTSRDEGs. This panel presents the systematic workflow for analyzing CTSRDEGs in cervical endocervical adenocarcinoma and squamous cell carcinoma (CESC) using The Cancer Genome Atlas (TCGA) data. It covers the steps to identify differentially expressed genes (DEGs) upon Cryptotanshinone (CTS) treatment, followed by subsequent functional enrichment analysis (Gene Ontology (GO) and Kyoto Encyclopedia of Genes and Genomes (KEGG) pathways), differential expression profiling (Exp Diff), and validation through Receiver Operating Characteristic (ROC) analysis and LASSO regression. Also shown are the methods for constructing the protein–protein interaction (PPI) network. **(B)** Cryptotanshinone and Targets Interaction Network. This panel illustrates the interaction network of CTS (yellow oval) with its predicted targets: DGIdb prediction targets (red circles), PubChem predicted targets (orange circles), and SwissTargetPrediction targets (purple circles). This network provides insights into the potential mechanisms of action of CTS in targeting gene expressions in CESC.

Bioinformatics analysis of cryptotanshinone is shown in Figure 1A. Cryptotanshinone (CTS) was used as a keyword to search the PubChem and DGIdb databases to identify the CTS-related targets. The target of CTS was predicted by SwissTargetPrediction website, and CTS-related targets identified by the three methods were combined to obtain 133 CTSRGs. The Cytoscape network diagram of CTS- and PPPPC-related genes (CTSRGs) is shown in Figure 1B. Detailed information is presented in Supplementary Table S2.

### 3.2 Differentially expressed genes associated with cryptotanshinone in cervical cancer

**FIGURE 2:**
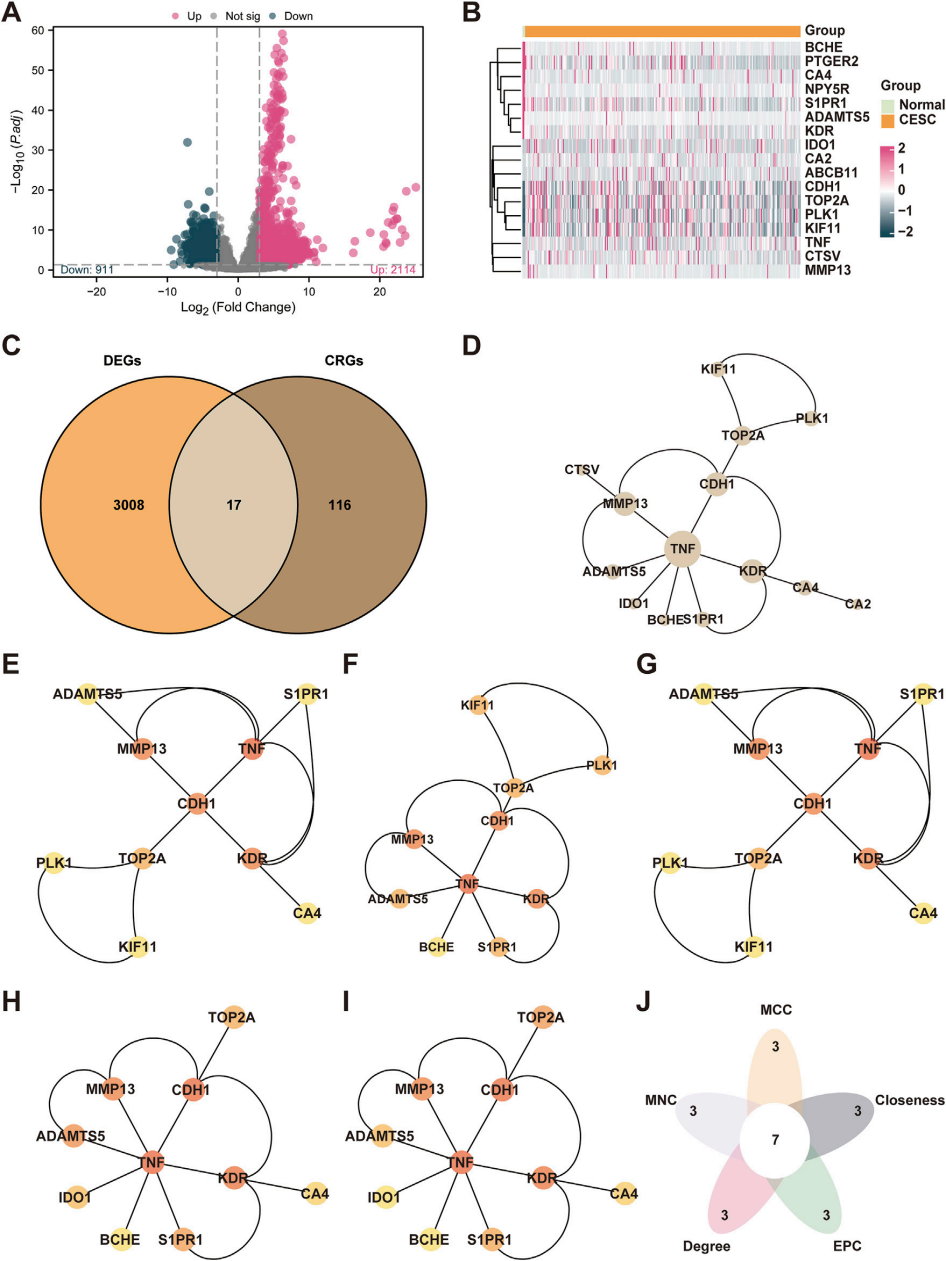
Integrated Analysis of Cryptotanshinone-Associated Gene Expression and Protein Interactions in Cervical Cancer. **(A)** Volcano plot illustrating the differential gene expression between the cervical cancer (CESC) group and the control (Normal) group in TCGA-CESC dataset. **(B)** Venn diagram showing the intersection of differentially expressed genes (DEGs) and cryptotanshinone-associated genes (CTSRGs) in cervical cancer. **(C)** Heat map of cryptotanshinone-associated differentially expressed genes (CTSRDEGs) showing gene expression levels in the cervical cancer dataset (TCGA-CESC), with high expression denoted by red and low expression by blue. **(D)** Protein–protein interaction (PPI) network of CTSRDEGs, analyzed utilizing the STRING database and visualized to highlight the top 10 hub genes as determined by five CytoHubba algorithms: MCC, MNC, Degree, EPC, and Closeness. **(E–I)** The PPI networks of the top 10 cryptotanshinone-related differentially expressed genes (CTSRDEGs) were constructed using five different algorithms from the CytoHubba plugin, revealing unique interactions among the genes. **(J)** The Venn diagram illustrates the overlap among the top genes identified by these algorithms in the context of cervical cancer. Color coding: orange represents the cervical cancer (CESC) group and green the control (Normal) group.

The dataset from The Cancer Genome Atlas (TCGA-CESC) was segregated into two groups: cases of cervical endocervical adenocarcinoma and squamous cell carcinoma (CESC) and normal controls. We conducted an analysis of differential gene expression between these groups utilizing the limma package in R.This analysis identified 3,024 differentially expressed genes, adhering to the criteria of an absolute log fold change (|logFC|) greater than 3.0 and an adjusted *p*-value (adj.P) below 0.05. Specifically, there was an upregulation of 2,114 genes and a downregulation of 911 genes under these conditions. We illustrated these findings in a volcano plot (Figure 2A).

In order to detect Cryptotanshinone-Related Differentially Expressed Genes (CTSRDEGs), we intersected the genes meeting the differential expression criteria with known Cryptotanshinone-Sensitive/Responsive Genes (CTSRGs). This intersection yielded 17 significant CTSRDEGs, including *TOP2A*, *PLK1*, *KIF11*, *CDH1*, *CTSV*, *ADAMTS5*, *S1PR1*, *KDR*, *CA2*, *BCHE*, *PTGER2*, *TNF*, *ABCB11*, *MMP13*, *NPY5R*, *CA4*, and *IDO1*. To visualize the expression patterns of these CTSRDEGs across the diverse groups within the TCGA-CESC dataset, we employed the pheatmap package in R to generate heat maps highlighting the differential expression (Figures 2B,C).

### 3.3 Construction of PPI networks and screening of hub genes

Initially, an analysis to discern protein protein interactions (PPI) was conducted, leading to the creation of a PPI network for 17 core CTSRDEGs utilizing the STRING database, as depicted in Figure 2D. This analysis led to the retention of key interacting CTSRDEGs. The PPI network revealed 14 connected CTSRDEGs: *MMP13*, *TNF*, *CA4*, *KDR*, *TOP2A*, *S1PR1*, *PLK1*, *ADAMTS5*, *BCHE*, *CA2*, *CDH1*, *CTSV*, *IDO1*, and *KIF11*. The relevance of these genes was further quantified using five distinct algorithms provided by the CytoHubba plugin in Cytoscape, ranking the genes based on their interaction scores. The algorithms employed were MCC, Degree, MNC, EPC, and Closeness. The PPI networks of the top 10 cryptotanshinone-related differentially expressed genes (CTSRDEGs) were constructed using five different algorithms from the CytoHubba plugin, revealing unique interactions among the genes (Figures 2E–I), where the gradation from red to yellow in the nodes denotes scores from high to low. A synthesis of the results from the five algorithms highlighted in Figure 2J identified seven hub genes critical to CESC: *TNF*, *MMP13*, *KDR*, *CDH1*, *TOP2A*, *ADAMTS5*, and *S1PR1*.

### 3.4 Enrichment analysis of GO and KEGG pathway

**FIGURE 3:**
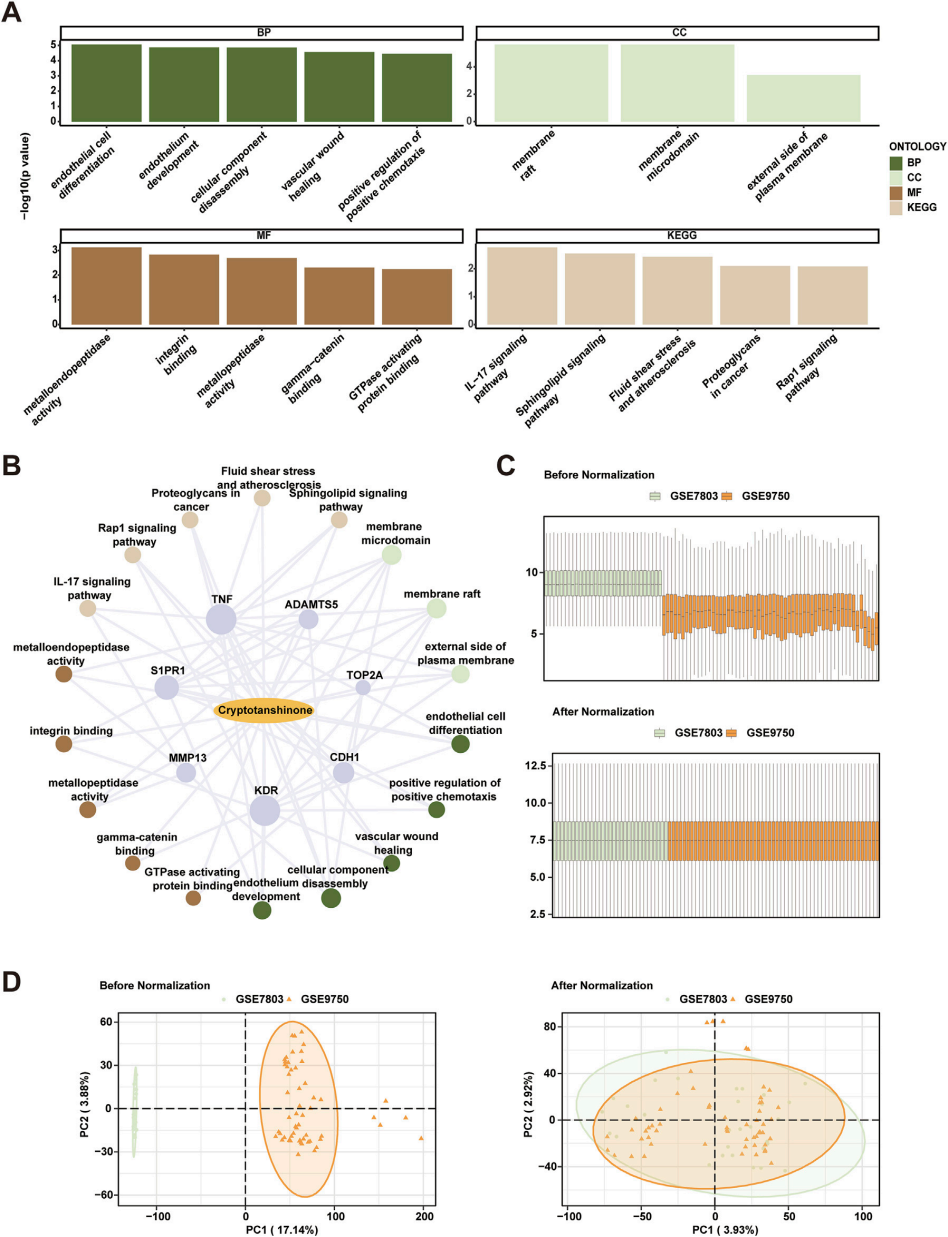
Comprehensive Analysis of Cryptotanshinone-Induced Gene Expression and Batch Effects Correction in Cervical Cancer Data Sets. **(A)** Gene Ontology (GO) and Kyoto Encyclopedia of Genes and Genomes (KEGG) pathway enrichment analyses of cryptotanshinone-related differentially expressed genes (CTSRDEGs) depicted via bubble maps for Biological Process (BP), Cellular Component (CC), Molecular Function (MF), and biological pathways (KEGG). The horizontal axes represent GO and KEGG terms. **(B)** Drug-target-pathway network illustrating interactions among cryptotanshinone (CTS), hub genes, GO entries, and KEGG pathways. The network uses yellow ovals for drugs, purple circles for genes, light brown for KEGG pathways, light green for CC entries, dark green for BP entries, and dark brown for MF entries. **(C)** Boxplot distribution of combined GEO datasets GSE7803 and GSE9750, illustrating batch effects before and after correction. **(D)** Principal Component Analysis (PCA) plots of the integrated GEO datasets, comparing data clustering before and after batch effect removal, with the cervical endocervical adenocarcinoma and squamous cell carcinoma (CESC) dataset GSE7803 in green and GSE9750 in orange. The screening criteria for GO and KEGG pathway enrichment analysis were *p*-value <0.05 and FDR value (q value) <0.25. In the network diagram, yellow ovals represent drugs, purple circles represent genes, light brown represents KEGG pathways, light green circles represent CC entries, dark green represents BP entries, and dark brown represents MF entries.

Subsequent GO and KEGG pathway analyses were employed to delineate the biological processes (BP), cellular components (CC), molecular functions (MF), and pathway involvements of the seven identified hub genes linked to cryptotanshinone. As illustrated in Table 2, these analyses elucidated significant enrichment of the hub genes across diverse biological processes, including endothelial cell differentiation, endothelium development, and vascular wound healing; cellular components including membrane raft and external side of plasma membrane; and molecular functions like metalloendopeptidase activity and integrin binding. Key pathways identified included the IL-17 signaling pathway, sphingolipid signaling pathway, and fluid shear stress and atherosclerosis, among others detailed in the Rap1 signaling pathway and proteoglycans in cancer. These results were visually represented in a bubble chart (Figure 3A) and further supported by a drug-target-pathway network diagram (Figure 3B). Additional comparisons in the KEGG enrichment analysis were made for the sphingolipid signaling pathway, as well as the fluid shear stress and atherosclerosis pathway, as shown in Supplementary Figures S1A, B. The visualization of these pathways was enhanced with the use of the R package Pathview for Supplementary Figures S1C–E.

**TABLE 2:** Results of GO and KEGG enrichment analysis for CTSRDEGs.

Ontology	ID	Description	BgRatio	*p*-value	p.adjust	qvalue
BP	GO:0045446	endothelial cell differentiation	121/18903	8.79E-06	4.75E-03	1.69E-03
BP	GO:0003158	endothelium development	139/18903	1.33E-05	4.75E-03	1.69E-03
BP	GO:0022411	cellular component disassembly	483/18903	1.39E-05	4.75E-03	1.69E-03
BP	GO:0061042	vascular wound healing	22/18903	2.71E-05	6.52E-03	2.32E-03
BP	GO:0050927	positive regulation of positive chemotaxis	25/18903	3.51E-05	6.52E-03	2.32E-03
CC	GO:0045121	membrane raft	326/19869	2.40E-06	4.12E-05	2.81E-05
CC	GO:0098857	membrane microdomain	327/19869	2.42E-06	4.12E-05	2.81E-05
CC	GO:0009897	external side of plasma membrane	462/19869	4.08E-04	4.62E-03	3.15E-03
MF	GO:0004222	metalloendopeptidase activity	112/18432	7.53E-04	3.09E-02	1.27E-02
MF	GO:0005178	integrin binding	156/18432	1.45E-03	3.09E-02	1.27E-02
MF	GO:0008237	metallopeptidase activity	184/18432	2.01E-03	3.09E-02	1.27E-02
MF	GO:0045295	gamma-catenin binding	13/18432	4.93E-03	3.68E-02	1.51E-02
MF	GO:0032794	GTPase activating protein binding	15/18432	5.68E-03	3.68E-02	1.51E-02
KEGG	hsa04657	IL-17 signaling pathway	94/8644	1.71E-03	1.10E-01	8.29E-02
KEGG	hsa04071	Sphingolipid signaling pathway	121/8644	2.81E-03	1.10E-01	8.29E-02
KEGG	hsa05418	Fluid shear stress and atherosclerosis	139/8644	3.69E-03	1.10E-01	8.29E-02
KEGG	hsa05205	Proteoglycans in cancer	205/8644	7.88E-03	1.44E-01	1.09E-01
KEGG	hsa04015	Rap1 signaling pathway	210/8644	8.26E-03	1.44E-01	1.09E-01

GO, gene ontology; BP, biological process; CC, cellular component; MF, molecular function; KEGG, kyoto encyclopedia of genes and genomes; CTSRDEGs, Cryptotanshinone-Related Differentially Expressed Genes.

### 3.5 Consolidation of cervical cancer datasets

Initially, the sva package in R was utilized to combine the datasets GSE7803 and GSE9750, aiming to rectify batch effects present within the CESC samples. The consistency of expression values pre- and post-adjustment was visually inspected using box plots (Figure 3C). Additionally, the dimensional reduction achieved through this correction was depicted using PCA, validating the effective alleviation of batch effects., as illustrated in Figure 3D.

### 3.6 Differential expression verification and ROC curve analysis of cryptotanshinone- related hub genes

**FIGURE 4:**
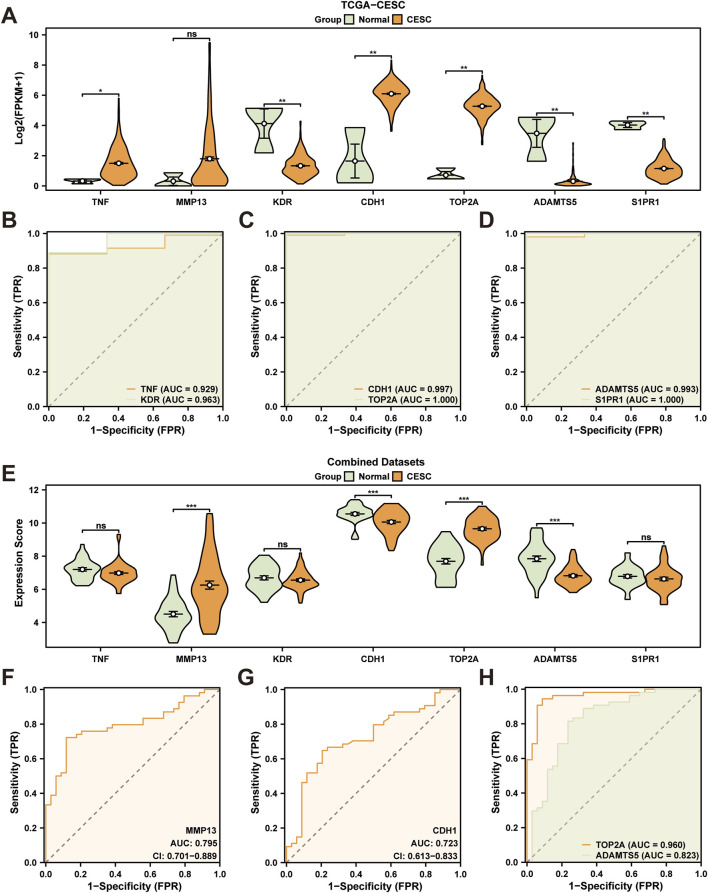
Differential Expression Validation and ROC Curve Analysis. **(A)** Grouping comparison diagram of cryptotanshinone-associated hub genes in cervical cancer (CESC) group and control (Normal) group in the Cervical cancer dataset (TCGA-CESC). **(B–D)** ROC curves of cryptotanshinone-associated hub genes *TNF* and *KDR* **(B)**, *CDH1* and *TOP2A* **(C)**, *ADAMTS5* and *S1PR1* **(D)** in TCGA-CESC dataset. **(E)** Grouping comparison diagram of cryptotanshinone-associated hub genes in cervical cancer (CESC) group and control (Normal) group in the integrated GEO dataset (Combined Datasets). **(F-H)** ROC curves of cryptotanshinone-associated hub genes *MMP13* **(F)**, *CDH1* **(G)**, *TOP2A*, and *ADAMTS5* **(H)** in an integrated GEO dataset (Combined Datasets). CESC, Cervical Endocervical Adenocarcinoma and Squamous Cell Carcinoma; CTSRDEGs, Cryptotanshinone-Related Differentially Expressed Genes; DCA, Decision Curve Analysis; ROC, Receiver Operating Characteristic; AUC, Area Under the Curve. ns indicates *p*-values ≥0.05, which were not statistically significant. *p < 0.05, statistical significance; **p < 0.01, highly statistically significant; ***p < 0.001, highly statistically significant. The AUC has high accuracy above 0.9, and the AUC has some accuracy between 0.7 and 0.9. Orange represents cervical cancer (CESC) group and green represents control (Normal) group.

We conducted an analysis to identify expression discrepancies of seven cryptotanshinone-sensitive hub genes within the CESC and control cohorts within the TCGA-CESC dataset, employing the Mann-Whitney U test. This analysis revealed statistically significant disparities (*p* < 0.05) for six of these genes: *TNF*, *KDR*, *CDH1*, *TOP2A*, *ADAMTS5*, and *S1PR1*, as shown in the comparative plot (Figure 4A). We then evaluated the diagnostic potential of these genes using ROC curve analyses, which demonstrated a high level of diagnostic accuracy (AUC > 0.9) in discriminating between the groups. (Figures 4B–D).

Following this, expression variances of the same hub genes were examined in the integrated GEO dataset, identifying significant differences for four genes: *MMP13*, *CDH1*, *TOP2A*, and *ADAMTS5* (*p* < 0.05), as documented in Figure 4E. ROC curves based on these genes demonstrated substantial diagnostic utility, with *TOP2A* showing high accuracy (AUC > 0.9) and *MMP13*, *CDH1*, and *ADAMTS5* exhibiting moderate accuracy (0.7 < AUC <0.9) for classifying CESC and normal samples (Figures 4F–H).

### 3.7 Construction and prognostic analysis of cervical cancer prognostic model

We utilized univariate Cox regression analysis, incorporating 17 CTSRDEGs, to devise a prognostic model specifically tailored for cervical squamous cell carcinoma and endocervical adenocarcinoma (CESC).

**FIGURE 5:**
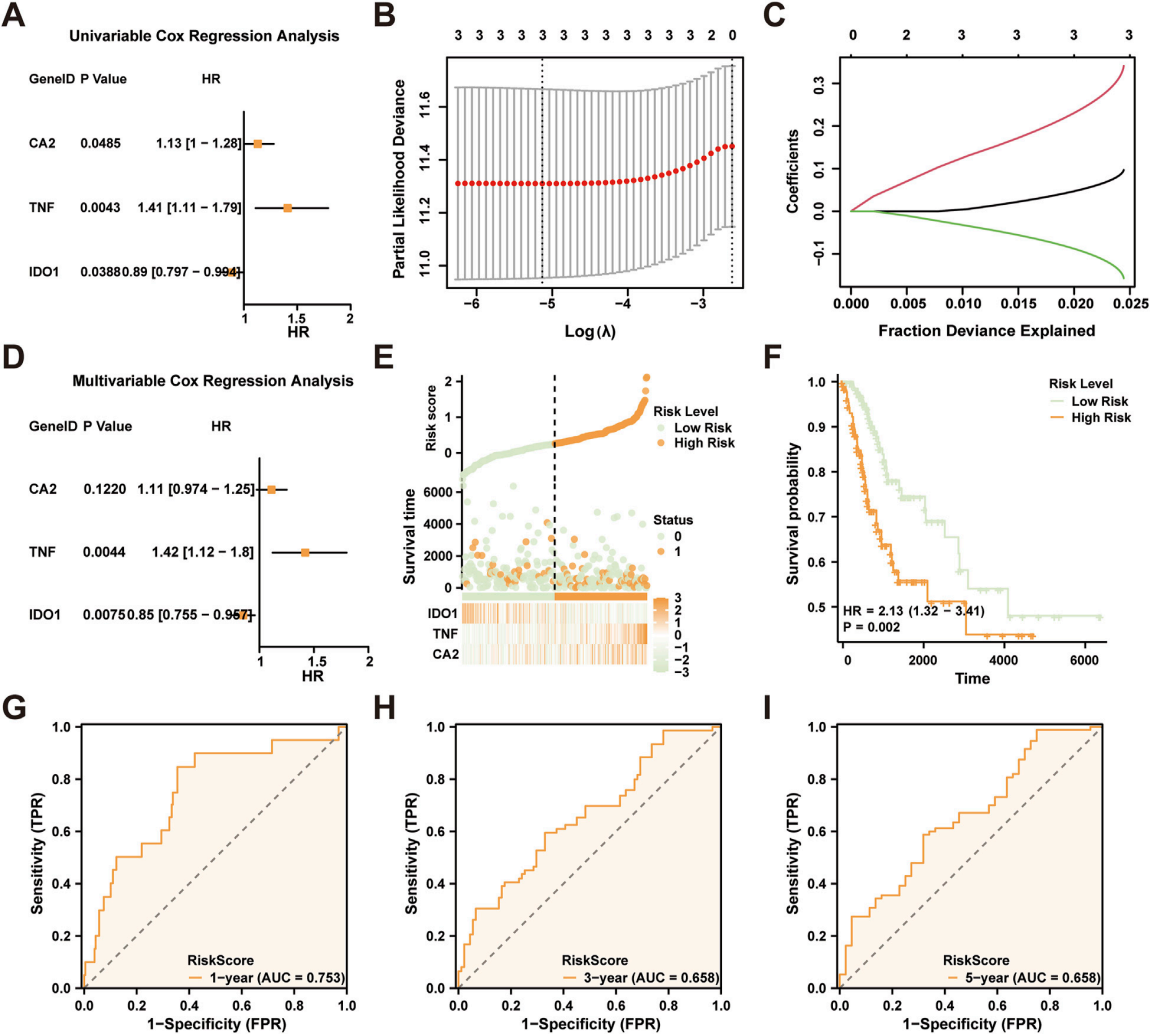
LASSO and Cox Regression Analysis. **(A)** Forest Plot of three cryptotanshinone-related differentially expressed genes (CTSRDEGs) in a univariate Cox regression model. The prognostic risk model plot **(B)** and variable locus plot **(C)** of the **(B, C)**. LASSO regression model. **(D)** Forest Plot of three prognostic risk model-associated genes in multifactor Cox regression model. **(E)** Risk factor plot of LASSO RiskScore **(F)** Prognostic KM curve between high and low groups of the LASSO risk Score (RiskScore) and overall survival (OS) of cervical cancer (CESC). G-I. 1 year **(G)**, 3 years **(H)**, and 5 years **(I)** LASSO risk scores depend on the ROC curve. TCGA, The Cancer Genome Atlas; CESC, Cervical Endocervical Adenocarcinoma and Squamous Cell Carcinoma; CTSRDEGs, Cryptotanshinone-Related Differentially Expressed Genes; LASSO, Least Absolute Shrinkage and Selection Operator; OS, Overall Survival; KM, Kaplan-Meier; ROC, Receiver Operating Characteristic Curve; AUC, Area Under the Curve. AUC has some accuracy at 0.7 to 0.9. The color green is employed to denote the low-risk group, whereas orange is utilized to signify the high-risk group.

Variables exhibiting a *p*-value less than 0.10 were subsequently illustrated in forest plots (Figure 5A). This analysis identified three significant CTSRDEGs: *CA2*, *TNF*, and *IDO1*. To refine our prognostic model, we applied LASSO regression to these genes, visualized using both a model diagram and a variable trace plot (Figures 5B,C). This step confirmed the inclusion of *CA2*, *TNF*, and *IDO1* in our LASSO model. Subsequent multivariate Cox regression analysis, focused on these genes, was conducted to explore their relationship with clinical outcomes and their prognostic efficacy. Results were depicted in another Forest Plot (Figure 5D). The formula for the LASSO RiskScore was established as:

$$\text{riskScore}=\text{CA}2*\;\left(0.0895\right)+\text{TNF}*\;\left(0.3250\right)+\text{IDO}1*\;\left(‐0.1470\right)$$

Using this score, we constructed a risk factor map (Figure 5E), employing the ggplot2 package, and stratified the CESC samples from TCGA-CESC dataset into high and low-risk categories based on the median RiskScore. The impact of this stratification on overall survival (OS) was assessed through the analysis of Kaplan-Meier (KM) curves, using the survival package (Figure 5F), and revealed significant differentiation in prognostic outcomes (*p* < 0.05). Time-dependent ROC curves for 1-, 3-, and 5-year forecasts were generated to assess the predictive accuracy of the RiskScore (Figures 5G–I), with the 1-year AUC reaching 0.753, indicating strong predictive power.

### 3.8 Validation of cervical cancer prognostic model

**FIGURE 6:**
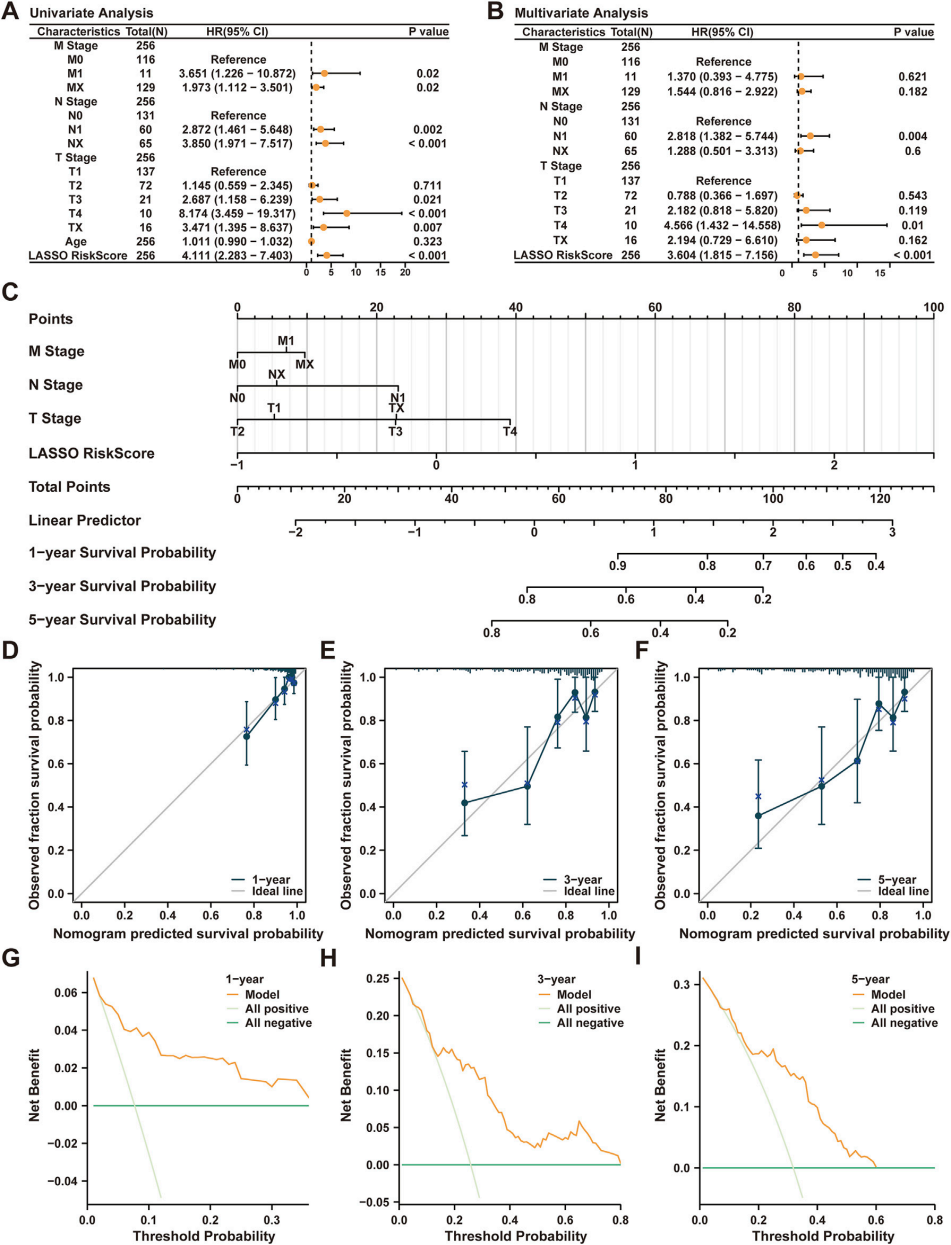
Validation of Prognostic Model. **(A)** Forest Plot of three clinical stage variables (T stage, N stage, M stage), Age, and LASSO RiskScore in univariate Cox regression model. **(B)** Forest Plot of three clinical stage variables (T stage, N stage, M stage), LASSO risk score (RiskScore) in multivariate Cox regression model. **(C)** Nomogram of three clinical stage variables (T stage, N stage, M stage) and LASSO RiskScore in a single multifactor Cox regression model. **(D-F)** 1-year **(D)**, 3-year **(E)**, and 5-year **(F)** calibration curves of CESC prognostic risk model; G-I. 1-year **(G)**, 3-year **(H)**, and 5-year **(I)** decision curve analysis (DCA) graph of cervical cancer (CESC) prognostic risk model. TCGA, The Cancer Genome Atlas; CESC, Cervical Endocervical Adenocarcinoma and Squamous Cell Carcinoma; LASSO, Least Absolute Shrinkage and Selection Operator; OS, Overall Survival.

The prognostic model’s reliability was further examined through calculations involving the LASSO RiskScore, based on gene expression levels and coefficients from the CESC dataset. Univariate Cox regression analyses were conducted utilizing the RiskScore, alongside age and stages (T, N, M), where all factors with *p*-values below 0.10 advanced to multivariate analysis (Table 3). The results, shown in forest plots (Figures 6A,B), affirmed the significance of the clinical stage variables and the RiskScore in predicting clinical outcomes. A nomogram integrating the RiskScore with T, N, and M stages was crafted to depict their prognostic relationships (Figure 6C), underscoring the superior prognostic value of the RiskScore over other variables. Calibration of the prognostic model at 1-, 3-, and 5-year intervals was performed, with calibration curves demonstrating close alignment with ideal predictions, especially at the 1-year mark (Figures 6D–F). Finally, the clinical usefulness of the model was assessed across different time frames through decision curve analysis (DCA). (Figures 6G–I). The analysis showed the model’s net benefit to be most substantial for predictions at 3 years, followed by 1 year, and 5 years, highlighting its effective prognostic capability across these intervals.

**TABLE 3:** Results of cox analysis.

Characteristics	Total (N)	Univariate analysis		Multivariate analysis	
		Hazard ratio (95% CI)	*p*-value	Hazard ratio (95% CI)	*p*-value
M Stage	256				
M0	116	Reference		Reference	
M1	11	3.651 (1.226–10.872)	0.020	1.370 (0.393–4.775)	0.621
MX	129	1.973 (1.112–3.501)	0.020	1.544 (0.816–2.922)	0.182
N Stage	256				
N0	131	Reference		Reference	
N1	60	2.872 (1.461–5.648)	0.002	2.818 (1.382–5.744)	0.004
NX	65	3.850 (1.971–7.517)	<0.001	1.288 (0.501–3.313)	0.600
T Stage	256				
T1	137	Reference		Reference	
T2	72	1.145 (0.559–2.345)	0.711	0.788 (0.366–1.697)	0.543
T3	21	2.687 (1.158–6.239)	0.021	2.182 (0.818–5.820)	0.119
T4	10	8.174 (3.459–19.317)	<0.001	4.566 (1.432–14.558)	0.010
TX	16	3.471 (1.395–8.637)	0.007	2.194 (0.729–6.610)	0.162
Age	256	1.011 (0.990–1.032)	0.323		
LASSO RiskScore	256	4.111 (2.283–7.403)	<0.001	3.604 (1.815–7.156)	<0.001

HR, hazard ratio, general HR > 1 indicates that the variable is a risk factor and HR < 1 is a protective factor. A single factor *p*-value <0.1 was included in the analysis.

### 3.9 Differential expression validation and correlation analysis in high- and low-risk groups

**FIGURE 7:**
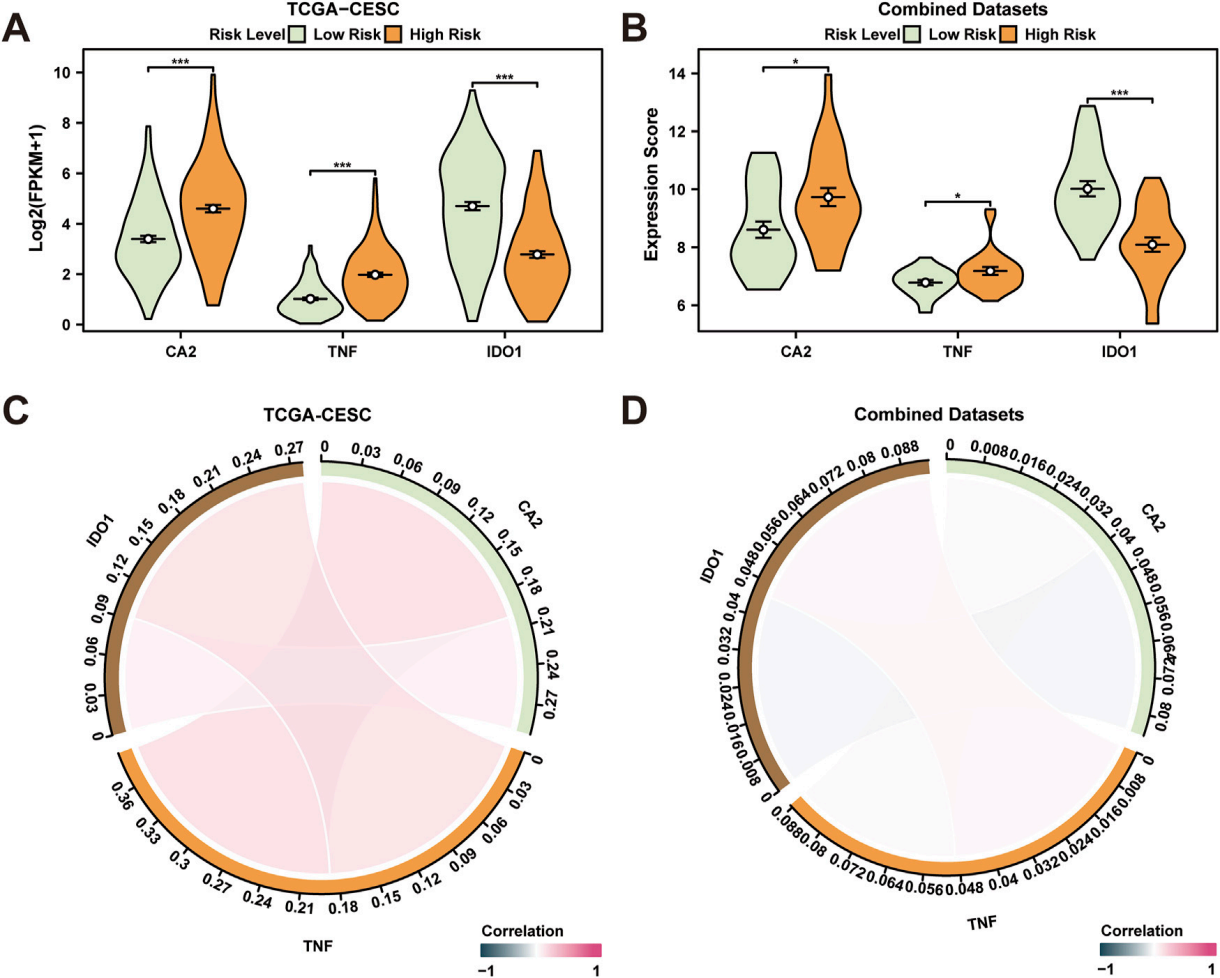
Differential Expression Validation and Correlation Analysis. **(A, B)** High risk of prognostic risk model-associated genes in cervical cancer (CESC) samples **(A)** from the cervical cancer dataset (TCGA-CESC) and cervical cancer (CESC) samples **(B)** from the Combined GEO Datasets (High) Subgroup comparison graph in the High-Risk group and the Low-Risk group. **(C, D)** Prognostic risk model-associated genes in cervical cancer (CESC) samples **(C)** from the Cervical cancer dataset (TCGA-CESC) and cervical cancer (CESC) samples **(D)** from the integrated GEO dataset (Combined Datasets). TCGA, The Cancer Genome Atlas; CESC, Cervical Endocervical Adenocarcinoma and Squamous Cell Carcinoma. *p < 0.05, which has statistical significance; ***p < 0.001, which is highly statistically significant. A correlation coefficient (r value) with an absolute value below 0.3 suggests weak or no correlation, while values between 0.3 and 0.5 indicate a weak correlation, those between 0.5 and 0.8 suggest moderate correlation, and those above 0.8 indicate strong correlation. Red and blue represent positive and negative correlation, respectively. Orange and green represent the cervical cancer (CESC) and control (Normal) groups, respectively.

CESC specimens from TCGA-CESC dataset were segregated into high and low-risk categories based on median LASSO RiskScore derived from the prognostic risk model for CESC. To investigate differential gene expression linked to the prognostic risk model, a comparison chart (Figure 7A) displayed variations in expression of three key genes associated with risk in both high- and low-risk CESC groups. Analysis revealed statistically significant differential expression for genes *CA2*, *TNF*, and *IDO1* across high- and low-risk groups (*p* < 0.001). Furthermore, CESC samples from the combined GEO dataset were categorized similarly using LASSO RiskScore. Differential expression of the same three genes in these groups was illustrated in a comparison figure (Figure 7B), indicating a highly significant variation in *IDO1* expression between high- and low-risk groups (*p* < 0.001), with *CA2* and *TNF* also showing significant differences (*p* < 0.05). A correlation analysis of these genes was conducted across samples from both TCGA-CESC and combined GEO datasets, visualized in a correlation chord diagram (Figures 7C,D). The analysis demonstrated a positive correlation between *IDO1* and *TNF*, and *CA2* and *TNF*, with no significant correlation observed between *IDO1* and *CA2*.

### 3.10 Immuno-infiltration analysis of cervical cancer

The TCGA-CESC dataset served as a basis for evaluating the correlation between 22 immune cell types and the classification of samples into CESC and normal groups utilizing the CIBERSORT algorithm. An immuno-infiltration histogram (Supplementary Figure S2A) depicted the proportions of immune cells, and differences in immune cell abundance between CESC and normal groups were highlighted in a subgroup comparison graph (Supplementary Figure S2B). Significant differences (*p* < 0.05) were found in 10 types of immune cells, including plasma cells, CD4^+^ T cells, resting memory CD4^+^ T cells, resting NK cells, monocytes, and various macrophage subsets among others. Correlation strengths among these immune cells were depicted in a heatmap (Supplementary Figure S2C), showing a strong positive correlation between resting dendritic cells and resting memory CD4^+^ T cells (r = 0.44), and a notable negative correlation between resting memory CD4^+^ T cells and macrophages M1 (r = −0.46).

Additionally, a correlation bubble map (Supplementary Figure S2D) detailed was employed to delve into the intricate relationships existing between critical prognostic genes and immune cell infiltration. The gene *IDO1* was positively correlated with macrophage M1 (r > 0.0, *p*-value < 0.05) and negatively correlated with resting memory CD4^+^ T cells (r < 0.0, *p* < 0.05).

### 3.11 Analysis of immune infiltration in high- and low-risk groups

We evaluated the immune cell infiltration in the cervical endocervical adenocarcinoma and squamous cell carcinoma (CESC) samples from TCGA-CESC dataset, employing the ssGSEA algorithm to quantify the presence of 28 distinct immune cell types within high- and low-risk CESC cohorts. Initial analyses demonstrated notable differences in the infiltration levels of 15 distinct immune cell types between these groups, as evidenced by a group comparison plot (Supplementary Figure S3A). This plot highlighted significant variances (*p* < 0.05) across several immune cells, including activated B cells, both CD4 and CD8 T cells (activated, central memory, and effector memory), activated dendritic cells, eosinophils, immature cells (B cells and dendritic cells), macrophages, myeloid-derived suppressor cells (MDSC), monocytes, natural killer cells, natural killer T cells, and neutrophils.

To further analyze these differences, we constructed correlation heat maps (Supplementary Figures S3B, C) to display the relationships among the immune cells within the defined risk groups. These maps showed predominantly strong positive correlations among the immune cell populations in both risk categories. Additionally, correlation bubble maps (Supplementary Figures S3D, E) were used to clarify the intricate connections between prognostic risk model genes and the abundance of immune cells in the CESC samples, segregated by risk group.

Moreover, to clarify the intricate connections between prognostic risk model genes and the varying abundances of immune cells in the CESC samples, segregated by risk category, correlation bubble maps (Supplementary Figures S3D, E) were utilized.

Notably, in the high-risk group, *IDO1* exhibited a substantial positive correlation with effector memory CD8^+^ T cells (r = 0.618*; p* < 0.05), whereas in the low-risk group, the strongest positive correlation was between *IDO1* and activated CD4^+^ T cells (r = 0.696, *p* < 0.05). These findings underscore the intricate associations between immune cell dynamics and the molecular underpinnings of risk stratification in CESC.

## 4 Discussion

This study analyzed cervical cancer network drugs, focusing on cryptotanshinone and its related genes. Data from TCGA and GEO databases were used to identify cryptotanshinone-related gene expression changes in cervical cancer samples. Using various bioinformatics tools and methods, such as DESeq2 for examining differential expression, the STRING database for protein–protein interaction networks, and Cox regression analysis for prognostic modeling, this study identified key genes and pathways associated with cervical cancer. We additionally explored the prognostic significance of these genes in cervical cancer and validated our findings through immuno-infiltration and survival analyses.

Comparative analysis with the existing literature reveals that our research offers unique perspectives on the anticancer effects of cryptotanshinone on cervical cancer. While previous research has primarily focused on the cytotoxic effects of cryptotanshinone against various cancer cell lines, our work focuses on the underlying molecular dynamics, uncovering specific gene expression and protein–protein interaction networks influenced by cryptotanshinone (Yen et al., 2019). Notably, our discovery of essential genes and pathways offers a more detailed mechanistic understanding of the anticancer activity of cryptotanshinone and establishes a foundation for future therapeutic strategies for cervical cancer treatment.

The mechanistic insights derived from our study highlight the biological significance of the identified hub genes and pathways, providing a deeper understanding of the anticancer effects of cryptotanshinone in cervical cancer. These hub genes and pathways are intricately involved in cellular processes, encompassing apoptosis, cell cycle regulation, and immune response modulation (Kim et al., 2018; Su et al., 2021; Luo et al., 2019). The influence of cryptotanshinone on these genes and pathways suggests a multi-targeted approach that disrupts cancer cell proliferation and survival, while enhancing immune surveillance against tumor cells. This comprehensive analysis revealed the potential molecular mechanisms by which cryptotanshinone exerts its therapeutic effects, offering promising directions for targeted cancer therapy development.

The IL-17 signaling pathway plays a key role in immune modulation within the tumor microenvironment, promoting inflammation, tumor cell survival, and immune evasion (PMID: 39219271, PMID: 36053326, PMID: 35376994). In cervical cancer, IL-17 upregulation may drive immune escape and tumor proliferation, making it a critical target in our study. Our findings suggest that cryptotanshinone may exert anti-tumor effects by modulating the IL-17 pathway, potentially inhibiting cancer cell growth and metastasis through the regulation of IL-17 expression and its downstream signals. Further exploration of these mechanisms may clarify cryptotanshinone’s therapeutic potential in targeting IL-17 in cervical cancer. Our findings highlight the promise of cryptotanshinone as a new and effective therapeutic choice for treating cervical cancer. By demonstrating the influence of cryptotanshinone on key molecular pathways and hub genes associated with tumor progression and immune evasion, our study suggests new approaches for more effective and targeted treatment (Shin et al., 2009; Wang et al., 2020). In addition, the identified biomarkers offer valuable prognostic tools, potentially enabling the development of personalized medical approaches that optimize treatment outcomes for patients with cervical cancer. This study paves the way for future clinical trials exploring the efficacy and safety of cryptotanshinone-based therapies in clinical settings.

Cryptotanshinone’s demonstrated efficacy in modulating key molecular pathways highlights its translational potential as an adjunctive or alternative therapy for cervical cancer. Current therapeutic approaches, primarily based on chemoradiotherapy, often face limitations such as adverse side effects, resistance, and limited efficacy in advanced-stage or recurrent cervical cancer. Compared to conventional therapies, cryptotanshinone offers a multi-targeted approach by simultaneously influencing apoptotic, proliferative, and immune-modulatory pathways, as observed in our study. These advantages underscore cryptotanshinone’s potential to enhance treatment outcomes while reducing the burden of side effects. Future research should prioritize clinical trials to validate cryptotanshinone’s safety and efficacy in clinical settings, with particular attention to its role in multi-drug regimens. Additionally, exploring optimal dosing strategies and delivery mechanisms for cryptotanshinone may further establish its position within cervical cancer treatment paradigms, potentially improving patient outcomes and offering a valuable tool in oncologic care.

However, certain limitations warrant further investigation. The small sample size in the TCGA-CESC dataset, which includes only three normal samples, may limit the generalizability of our findings. This constraint highlights the need for validation using larger and more diverse cohorts to enhance the robustness and applicability of the results in broader clinical settings. Future studies should aim to address this limitation by incorporating multi-center data or additional high-throughput datasets. Additionally, Our bioinformatics analysis suggests that cryptotanshinone may interact with multiple signaling pathways related to the inhibition of cancer cell proliferation and induction of apoptosis. However, as these conclusions are primarily based on computational data, further experimental studies are necessary to explore and validate the specific mechanisms of cryptotanshinone’s action. These limitations highlight the need for further research involving larger, more diverse cohorts, and the application of more comprehensive analytical methods to validate and extend our findings. Future studies should clarify the detailed molecular mechanisms through which cryptotanshinone acts on cancer cells, particularly by exploring its interaction with the novel targets identified in our study. Expanding our understanding of the pharmacodynamics of this drug and optimizing its delivery mechanisms could significantly enhance its clinical application and pave the way for novel targeted treatments for cervical cancer.

## 5 Conclusion

This report presents a comprehensive analysis of cervical cancer and its associated genes, with a specific focus on the impact of cryptosalvianols. Using TCGA and GEO databases, we identified DEGs in cervical cancer and employed bioinformatic tools to analyze their roles and prognostic value. The objective of this study was to lay the foundation for the advancement of targeted therapeutic approaches in the treatment of cervical cancer.

## Data Availability

The datasets presented in this study can be found in online repositories. The names of the repository/repositories and accession number(s) can be found in the article/Supplementary Material.

## Supplementary material

SUPPLEMENTARY FIGURE S1 Pathway of Cryptotanshinone-associated hub genes (KEGG) enrichment analysis and Pathway visualization. Sphingolipid signaling pathway **(A)**, Fluid shear stress and atherosclerosis **(B)**, IL-17 signaling pathway **(C)**, Proteoglycans in cancer **(D)** and Rap1 signaling pathway **(E)**. KEGG, Kyoto Encyclopedia of Genes and Genomes.

SUPPLEMENTARY FIGURE S2 TCGA-CESC Immune Infiltration Analysis using the CIBERSORT Algorithm. **(A, B)** Histogram **(A)** and group comparison graph **(B)** of immune infiltration in cervical cancer data set (TCGA-CESC). **(C)** Correlation heatmap depicting the levels of immune cell infiltration in the TCGA-CESC cervical cancer dataset (TCGA-CESC). **(D)** Bubble map of correlation between prognostic risk model genes and abundance of immune cell infiltration in the cervical cancer dataset (TCGA-CESC). TCGA, The Cancer Genome Atlas; CESC, Cervical Endocervical Adenocarcinoma and Squamous Cell Carcinoma. ns indicates *P*-values ≥0.05, which were not statistically significant. **P* < 0.05, statistical significance; ***P* < 0.01, highly statistically significant; ****P* < 0.001, highly statistically significant. The absolute value of the correlation coefficient (r value) between 0.3 and 0.5 indicates a weak correlation, and between 0.5 and 0.8 indicates a moderate correlation. Orange is the cervical cancer (CESC) group, green is the control (Normal) group. Blue is negatively correlated, and green is positively correlated.

SUPPLEMENTARY FIGURE S3 Risk Group Immune Infiltration Analysis using the ssGSEA Algorithm. **(A)** A comparative analysis of immune cells in High-Risk and Low-Risk groups of cervical cancer (CESC) samples. **(B, C)** Results of correlation analysis of the abundance of immune cell infiltration in the High-Risk group **(B)** and Low-Risk group **(C)** samples of cervical cancer (CESC). **(D, E)** Bubble chart depicting the relationship between immune cell infiltration levels and prognostic risk model genes in the High-Risk **(D)** and Low-Risk **(E)** groups of cervical cancer (CESC). ssGSEA, single-sample Gene-Set Enrichment Analysis; CESC, Cervical Endocervical Adenocarcinoma and Squamous Cell Carcinoma. ns indicates *P*-values ≥0.05, which were not statistically significant. **P* < 0.05, statistical significance; ***P* < 0.01, highly statistically significant; ****P* < 0.001, highly statistically significant. The absolute value of the correlation coefficient (r value) between 0.3 and 0.5 indicates a weak level of correlation, while values between 0.5 and 0.8 suggest a moderate degree of correlation. Orange is the High-Risk group, green is the Low-Risk group. Red is positively correlated, and blue is negatively correlated.
