# Supplementary material for: Exploring the therapeutic potency of cryptotanshinone in cervical cancer: a multi-omics and network pharmacology approach
Source: Front Genet. 2024 Nov 27;15:1435132. doi: 10.3389/fgene.2024.1435132 (PMC11632102; doi:10.3389/fgene.2024.1435132)
Supplement: Supplementary file 2 [file Table1.docx]

**Table S1 GEO Microarray Chip Information**

|  | GSE7803 | GSE9750 |
| --- | --- | --- |
| Platform | GPL96 | GPL96 |
| Species | Homo sapiens | Homo sapiens |
| Tissue | Cervix | Cervix |
| Samples in CESC group | 21 | 33 |
| Samples in Normal group | 10 | 24 |
| Reference | PMID：17974957 | PMID：18506748 |

GEO，Gene Expression Omnibus；CESC，Cervical Endocervical Adenocarcinoma and Squamous Cell Carcinoma。
