# Supplementary figures and images for: Exploring the therapeutic potency of cryptotanshinone in cervical cancer: a multi-omics and network pharmacology approach
Source: Front Genet. 2024 Nov 27;15:1435132. doi: 10.3389/fgene.2024.1435132 (PMC11632102; doi:10.3389/fgene.2024.1435132)

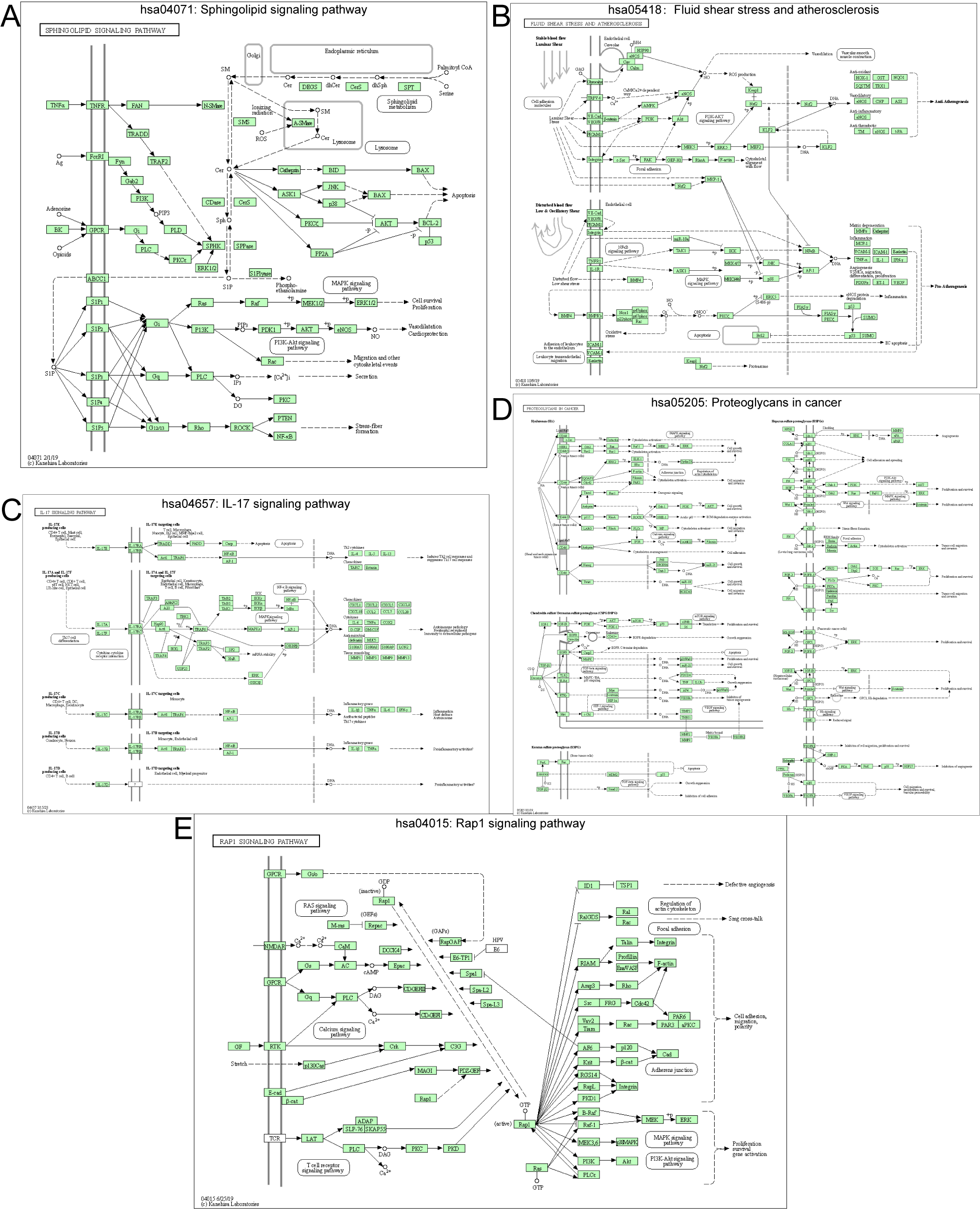

Supplement: Supplementary file 5 [file Image1.tif]
